# Pd(II)-Catalyzed C-H Acylation of (Hetero)arenes—Recent Advances

**DOI:** 10.3390/molecules25143247

**Published:** 2020-07-16

**Authors:** Carlos Santiago, Nuria Sotomayor, Esther Lete

**Affiliations:** Departamento de Química Orgánica II, Facultad de Ciencia y Tecnología, Universidad del País Vasco/Euskal Herriko Unibertsitatea UPV/EHU. Apdo. 644. 48080 Bilbao, Spain; carlos.santiago@ehu.es

**Keywords:** C-H activation, acylation, palladium, arenes, heteroarenes

## Abstract

Di(hetero)aryl ketones are important motifs present in natural products, pharmaceuticals or agrochemicals. In recent years, Pd(II)-catalyzed acylation of (hetero)arenes in the presence of an oxidant has emerged as a catalytic alternative to classical acylation methods, reducing the production of toxic metal waste. Different directing groups and acyl sources are being studied for this purpose, although further development is required to face mainly selectivity problems in order to be applied in the synthesis of more complex molecules. Selected recent developments and applications are covered in this review.

## 1. Introduction

Transition-metal direct C-H functionalization has emerged as an efficient, atom-economical and environmentally friendly synthetic tool for the preparation of complex multifunctional molecules, which is a good alternative to traditional cross-coupling chemistry. However, this transformation represents a significant challenge in chemical synthesis, largely due to the difficulties associated with the chemoselective activation of relatively inert C-H bonds. Palladium (II) catalysis has been widely used in C-H activation and functionalization of C(sp^2^)-H bonds of arenes, heteroarenes or even simple alkenes [1,2]. In this context, Pd(II)-catalyzed acylation of arenes in the presence of an oxidant has emerged as an interesting tool for the synthesis of di(hetero)aryl ketones, important motifs present in natural products, pharmaceuticals or agrochemicals. This procedure constitutes a catalytic alternative to classical acylation methods, reducing the production of toxic metal waste, although it still requires the use of a stoichiometric amount of an oxidant and, in some cases, additives such as acids or metal salts.

**Scheme 1:**
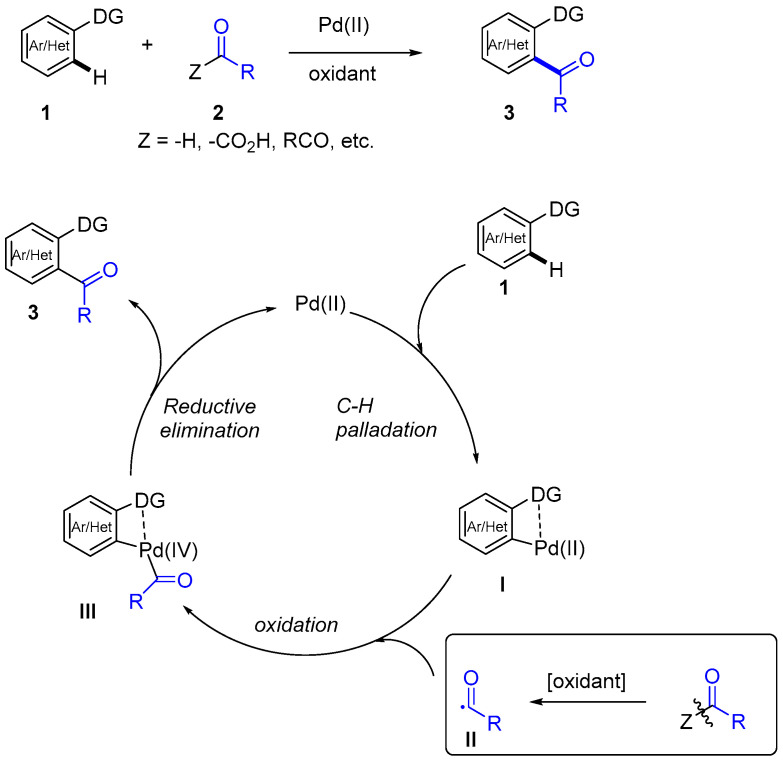
Schematic mechanistic proposal for Pd(II)-catalyzed acylation via C-H activation.

A schematic general mechanism proposal is depicted in Scheme 1 that implies three distinct fundamental steps. First, palladation of the arene or heteroarene **1** occurs to afford an arylpalladium(II) intermediate **I**, via C-H activation. The regioselectivity of this step is generally controlled by the use of a directing group (DG) [3] that provides the *ortho*-arylpalladium(II) intermediate **I**. On the other hand, the oxidant promotes de formation of an acyl radical **II** that, upon reaction with **I,** forms a Pd(IV) intermediate **III** after oxidation. Reductive elimination would afford the ketone **3**, recovering the Pd(II) catalyst. The first example of Pd(II)-catalyzed acylation reaction with aldehydes was reported in 2009 by Cheng using 2-pyridine as directing group [4]. Since then, significant advances have been made in the field, by the assistance of a wide variety of directing groups for the metalation (DG in **1**) and by the practical use of different precursors **2** for the acyl radical. The scope of the reaction is wide regarding the both the (hetero)aromatic ring **1** and the acyl radical equivalent **2**. The use of the directing group on **1** allows the reaction to proceed with both electron-donating and electron-withdrawing substituents on the aromatic ring, although better reactivity is generally obtained with electron-rich aromatic rings. Regarding the acyl radical equivalents **2**, in most cases aoryl groups are (ArCO) are introduced. Higher reactivities are generally observed when the aromatic ring of the acyl radical equivalent **2** is substituted with electron donating groups, what would be in accordance with a more nucleophilic radical intermediate **II**. The use aliphatic acyl equivalents, although possible, generally provides much lower yields. The use of peroxides, such as *t*-butylhydroperoxide (TBHP), as oxidants for the formation of the acyl radical is the most general, although more recently their generation via photoredox catalysis [5] has also been studied for these reactions.

Although most reactions take place according to Scheme 1, the actual mechanism is still not clear. For instance, the exact nature of species **III** formed after radical addition is not known in most cases. Density Functional Theory (DFT) calculations [6] support that the oxidative addition of an acyl radical, obtained by TBHP hydrogen atom abstraction from an aldehyde, to an arylpalladium (II) species would generate a Pd(IV) intermediate through a very exergonic process. Reductive elimination would lead to the acylated compound. An alternative aldehyde insertion mechanism was found to be unfavorable. Kinetic isotopic effect experiments have also been used to try to establish the rate-determining step. However, the results obtained for the K_H_/K_D_ ratio in some cases support that the C-H bond cleavage is involved in the rate-determining step [6] but not in other cases [7]. Alternatively, binding of the substrate with palladium and the formation of an aroyl palladium π complex has been proposed [7]. Consequently, further studies are required for mechanistic understanding and further development is necessary for this type of reactions in order to be applied in the synthesis of more complex molecules. A review appeared in 2015 covering the major achievements in this field [8] and the topic has also been included in a broader review [9]. Therefore, the present article will not attempt to provide exhaustive coverage of the literature but it is intended to focus on significant recent advances in this type of Pd(II) catalyzed reactions in arenes and heteroarenes and their applications. 

## 2. Acylation of Arenes 

**Scheme 2:**
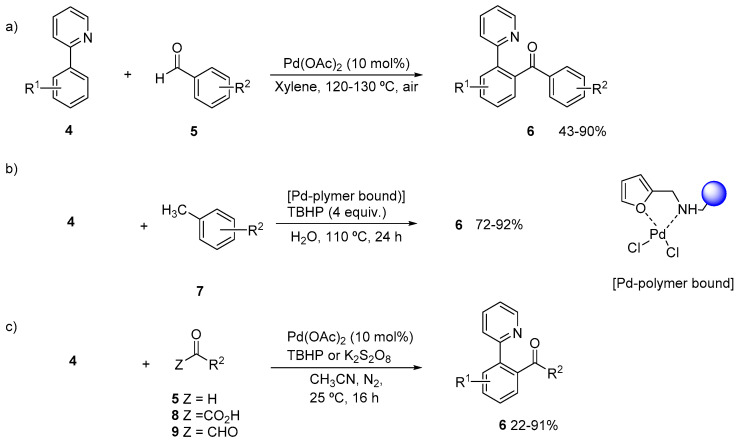
Acylation of arenes using a pyridine-directing group.

As indicated, the first example of Pd(II)-catalyzed acylation reaction was reported using 2-pyridine as directing group, aldehydes as the acyl precursor, although an aldehyde insertion mechanism was proposed in a Pd(II)/Pd(0)/Pd(II) cycle, using air as the oxidant [4] (Scheme 2a). Later, the use of TBHP as an oxidant was reported and a Pd(II)/Pd(IV)/Pd(II) cycle was proposed [10]. Homogeneous conditions are generally used for these reactions, using standard commercial Pd(II) pre-catalysts, such as Pd(OAc)_2_, Pd(TFA)_2_, PdCl_2_(CH_3_CN)_2_, sometimes in the presence of additives. However, it has been shown that a recyclable heterogeneous palladium complex under on-water conditions, using toluenes as the acyl source (Scheme 2b), can replace homogeneous catalysts. Polymer supported furan-2-ylmethanamine complex is stable and could be reused for 5 cycles without loss of efficiency [11]. α-Oxoacids have been also used as the acyl source in decarboxylative acylation reactions in the presence of silver salts [12]. Generally these reactions require the use of high temperatures (80–140 °C) but more recently it has been shown that aliphatic and aromatic aldehydes **5**, α-oxoacids **8** and α-oxoaldehydes **9** can be reacted at room temperature in CH_3_CN, in presence of Pd(OAc)_2_ as catalyst and K_2_S_2_O_8_ (for **8** and **9**) or TBHP (for aldehydes **5**) as oxidants. The formation of an acyl radical intermediate is proposed in all cases. Generally, good yields of **6** are obtained, although aldehydes required longer reaction times (36 h) (Scheme 2c) [13]. Interestingly, when 2-pyrimidine was used as directing group instead, mixtures of mono- and diacylated products were obtained. 

**Scheme 3:**
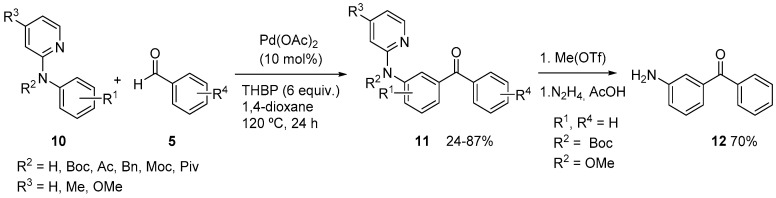
Pyridine as removable directing group. Effect of the substitution.

If the pyridine-directing group is linked to the aromatic ring through a heteroatom [7], it can be easily removed, so further transformations can be performed on the acylated compounds, significantly increasing the synthetic applicability of these transformations. Along these lines, the effect of the substitution on the pyridine ring was studied in the acylation of *N*-aryl-2-pyridinylamines **10** (Scheme 3) [14].

**Scheme 4:**
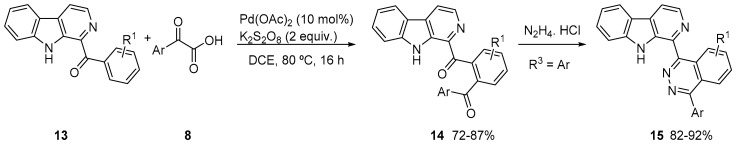
β-Carboline as directing group.

The introduction of an electron donating group in C-4 of the pyridine ring (R^3^ = OMe) promotes the reactivity of the Pd(II)-catalyzed C-H activation step. On the other hand, the amino nitrogen has to be protected (R^2^ = Boc, Ac, Bn, Moc, Piv) and Boc was selected as the most effective group. The reaction was extended to a series of aldehydes obtaining **11** in moderate to good yields, although in most cases significant amounts of the diacylated products were also obtained. Both protecting groups could be efficiently removed to obtain amine **12**, which could also be transformed into an acridanone [14]. Besides the use of simple 2-pyridinyl, other related directing groups based on nitrogen coordination have been developed. As shown in Scheme 4, β-carboline was used as directing group for the selective acylation of ketones **13** with α-oxoacids **8** using K_2_S_2_O_8_ as oxidant. Diketones **14** were obtained in good yields, which could be derivatized to phthalizines **15**. In this case, the pyridine nitrogen would facilitate the metalation by the formation of a six-membered palladacycle [15].

**Scheme 5:**
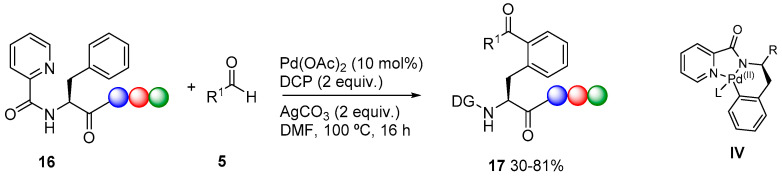
Late stage functionalization of peptides using a picolinamide directing group.

A very interesting application has been developed for the late stage functionalization of peptides, as shown in Scheme 5. Using a picolinamide directing group it was possible to carry out the selective acylation of phenylalanine-containing peptides **16** (from dipeptides to pentapeptides). The selective palladation would be favored by the bidentate directing group through the formation of a palladacycle intermediate such as **IV** that would later react with the acyl radical to generate a Pd(IV) intermediate. Aromatic, heteroaromatic and aliphatic aldehydes **5** could be used, using dicumyl peroxide (DCP) as oxidant in the presence of silver carbonate (TBHP gave lower yields). Under these conditions, generally excellent yields of the selectively monoacylated peptides **17** were obtained, minimizing the formation of diacylated compounds and with complete retention of stereochemistry [16].

**Scheme 6:**
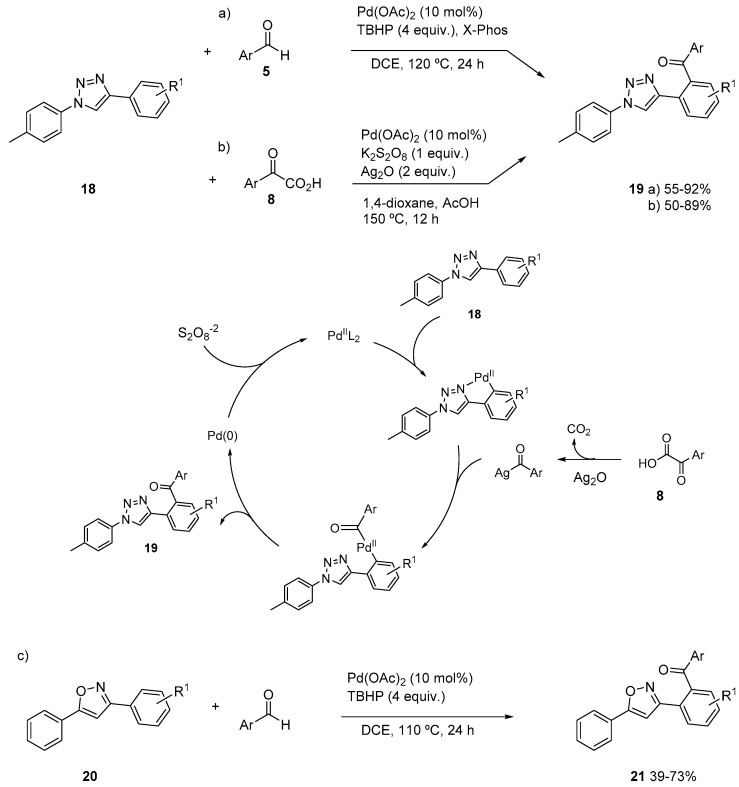
1,2,3-triazole and oxazole as directing groups.

Besides pyridine, other nitrogen heterocycles have been used as directing groups, such as triazoles The acylation of 2-aryl-1,2,3-triazoles has been accomplished with aldehydes [17]. More recently, when 1,4-diaryl-1,2,3-triazoles **18** were used, the acylation using aldehydes **5** [18] or oxoacids **8** [19] took place selectively on the aromatic ring on C-4 of the triazole (Scheme 6). In the first case (Scheme 6a), the yields of **19** were improved in the presence of a ligand such as X-Phos. The reaction using oxoacids **8** in the presence of silver oxide (Scheme 6b) does not seem to involve the formation of an acyl radical intermediate, as no change in reactivity was found when the reactions were carried out in the presence of radical scavengers such as 2,2,6,6-tetramethylpiperidin-1-yl-oxydanyl (TEMPO). A mechanism (Scheme 6) is proposed in which coordination to the more electron rich N-3 atom of the triazole would favor the *ortho*-palladation. The oxoacid **8** would generate an acylsilver intermediate, which would transmetalate to generate an acylpalladium(II) intermediate. Reductive elimination produces **19** and the resulting palladium(0) species is reoxidized to Pd(II), closing the catalytic cycle. In addition, the higher directing ability of nitrogen over oxygen atom has been applied for the completely regioselective acylation of 3,5-diarylisoxazoles **20** with aldehydes, which are acylated at the *ortho*-position of the C-3 aromatic ring to obtain **21** [20] (Scheme 6c). 

**Scheme 7:**
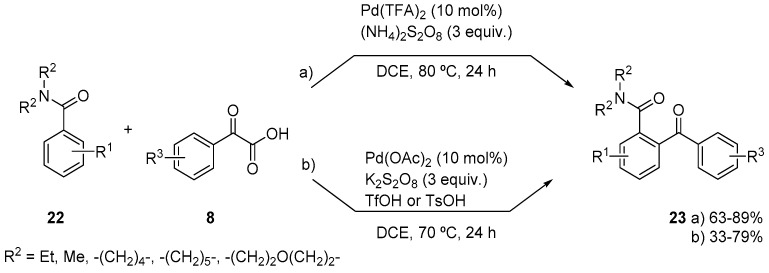
Tertiary amides as directing groups. Acylation of benzamides.

The use of tertiary amides as directing groups in *ortho*-metalation reactions is widely recognized and employed [21,22]. Diethyl amides have been used in rhodium-catalyzed aroylation reactions with aldehydes [23]. However, as tertiary amides may undergo insertion of palladium into the N-C(O) bond [24], they have been used as aroyl sources in cross-coupling reactions. Besides, the weak coordination of the amide and the electron deficient aryl ring makes them difficult substrates for palladium catalyzed C-H activation. Despite this, two examples of decarboxylative acylation of benzamides **22** with α-oxocarboxylic acids **8** were described almost simultaneously (Scheme 7) [25,26]. In both cases, dialkyl or cyclic amides could be used as directing groups. Diethyl amides (R^2^ = Et) were selected as the best candidates in the first case (Scheme 7a, [25]) using (NH_4_)_2_S_2_O_8_ as oxidant, while dimethyl amides (R^2^ = Me) were selected for extension in the second case (Scheme 7b, [26]). The mode of activation of the amide is not clear. Control experiments suggest that the initial palladation is assisted by initial coordination to the amide nitrogen, although DFT calculations showed that an O-coordinated intermediate would be more favorable [25]. Besides, intermolecular KIE experiments (K_H_/K_D_ = 2.5) suggest that the C-H cleavage might be the rate determining step. In this case, O-coordination to the amide is proposed [26].

**Scheme 8:**
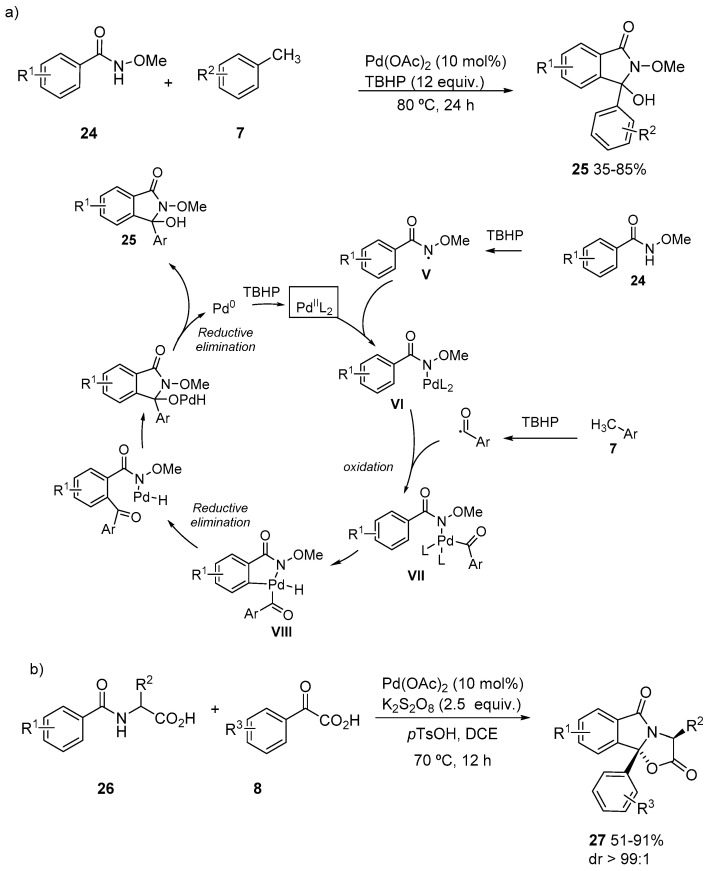
Secondary amides as directing groups. Formation of isoindolinones.

Secondary amides, with a free NH group, have also been successfully used as directing groups in the acylation reaction with aldehydes [27] or α-oxoacids [26], leading to the formation of hydroxyisoindolones through acylation and subsequent cyclization. More recently, toluene derivatives **7** have been used as acyl source for the acylation of *N*-methoxybenzamides **24** (Scheme 8a) [28]. 

In this case, the mechanistic proposal differs from Scheme 1, although a coupling with an acyl radical is also involved. In agreement with previous reports [27], it is proposed that the secondary amide would be activated by TBHP, generating an amide nitrogen radical **V**, which forms intermediate **VI** after electrophilic palladation. TBHP also oxidizes the toluene **7** to the acyl radical, forming a high-valent Pd(IV) intermediate **VII** prior to a fast C-H activation. Hydroxyindolinones **25** would be obtained through reductive elimination and subsequent cyclization, as depicted in Scheme 8a. Amino acid-derived amides have also been used as bidentate directing groups (Scheme 8b) [29]. The mechanistic proposal is analogous to the one depicted in Scheme 8a, although in this case, C-H activation is assisted by prior coordination of Pd(II) to both the nitrogen atom and the carboxylic oxygen atom forming a palladacycle intermediate. The directing group on benzamides **26** is incorporated in the oxazoloisoindolinones **27** through a cascade reaction, with complete diastereoselectivity.

**Scheme 9:**
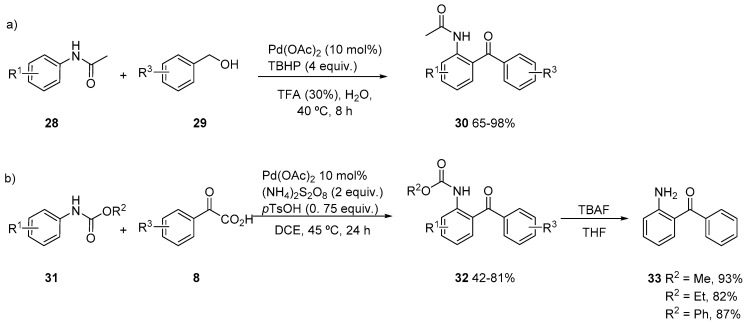
Acylation of acetanilides and *N*-aryl carbamates.

Besides benzamides, anilides have also been successfully acylated with aldehydes [30,31,32]. Also, an amide-directed metalation was applied in the selective C-7 acylation of indolines with aldehydes [33]. More recently, acylation of acetanilides **28** has been achieved with inexpensive benzylic alcohols **29** under aqueous conditions at 40 °C in the presence of a catalytic amount of TFA, obtaining very good yields of ketones **30**, with wide functional group tolerance (Scheme 9a) [34]. A bimetallic palladium 6-membered cyclopalladated complex could be obtained and used as catalyst for this reaction. Recently, carbamates have also been used as directing groups for the acylation with α-oxoacids **8** (Scheme 9b) obtaining **32** in moderate to good yields [35]. Carbamate directing group could be easily removed obtaining amine **33** that could be derivatized to various heterocyclic systems, such as quinazoline or phenylquinoline. 

**Scheme 10:**
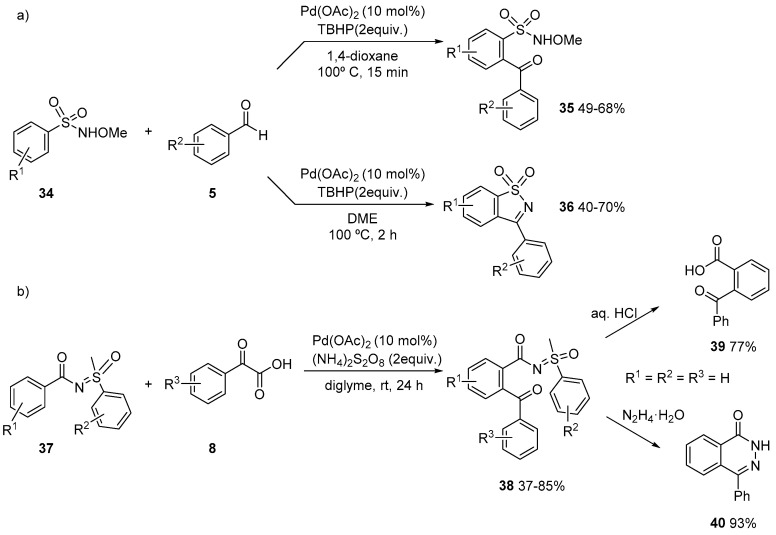
Acylation of sulfonamides and *N*-sulfoximine amides.

More recently, sulfonamides an *N*-sulfoximine amides have also been developed as directing groups (Scheme 10). Sulfonamides **34** are acylated efficiently with aldehydes **5** in good yields in only 15 min [36]. Aliphatic aldehydes can also be used, although with lower yields. Only a change in the solvent and an extension of the reaction time led to the direct formation of cyclic sulfonyl ketimines **36** in generally good yields (Scheme 10a). In the case of the *N*-sulfoximine benzamides **37**, the reaction could be efficiently performed with α-oxocarboxylic acids **8** at room temperature. The reactivity was similar when electron withdrawing and electron donating groups were introduced in the sulfoximine unit. (Scheme 10b). The directing group could be easily hydrolyzed to obtain the corresponding carboxylic acid **39** or directly transformed in other heterocyclic systems, such as phthalazinone **40 [37]**. 

**Scheme 11:**
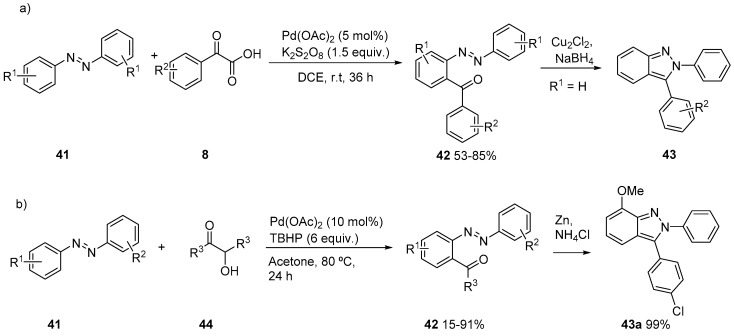
Acylation of azoarenes.

Besides these examples, other nitrogen containing directing groups have been also used. For instance, azobenzenes **41** have been selectively *ortho*-acylated with aldehydes [38], also using aqueous conditions [39] and with α-oxoacids **8** [40], even at room temperature [41]. The acylated compounds **42** could be very efficiently transformed into indazoles **43** (Scheme 11a). More recently, α-hydroxyl ketones **44** have been identified as acylating agents (Scheme 11b). In most examples, symmetrically substituted azoarenes are used, leading to the selective formation of monoacylated compounds, although excellent selectivities could also be obtained when non-symmetrically substituted azoarenes **41** were used, depending on the substitution pattern. This acylation reaction could be applied to the synthesis of a liver(X) receptor agonist **43a**. It is proposed that the hydroxyketone is oxidized to the corresponding diketone, generating the acyl radical. Both acyl fragments are transferred, so symmetrically substituted **44** have to be used to obtain selective reactions [42]. 

**Scheme 12:**
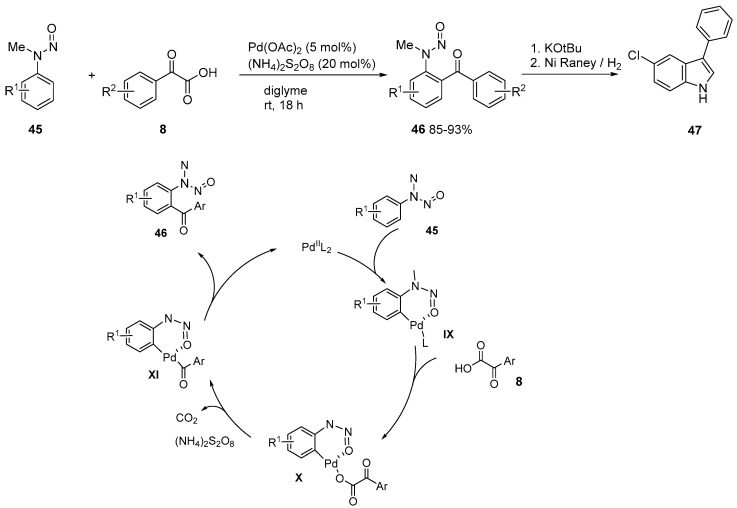
Nitrosoamine as directing group.

Nitrosoamines are also effective directing groups (Scheme 12). Thus, nitrosoanilines **45** could be acylated with oxoacids **8** at room temperature, using 5 mol % of the palladium catalyst. Ketones **46** are obtained in good yields, with wide functional group tolerance. The nitroso group could be removed or transformed, as exemplified in Scheme 12 for the two step synthesis of indole **47** [43]. Related examples have also been described [44,45]. The mechanism in these cases is still not clear but the formation of an intermediate acyl radical from the ketoacid **8** is not proposed. Instead, palladation of nitrosoanilines **45** lead to an intermediate **IX**, which would react with ketoacid **8** to obtain a Pd(II)carboxylate such as **X**. Decarboxylation would lead to an acyl-palladium(IV) intermediate **XI** after oxidation. 

**Scheme 13:**
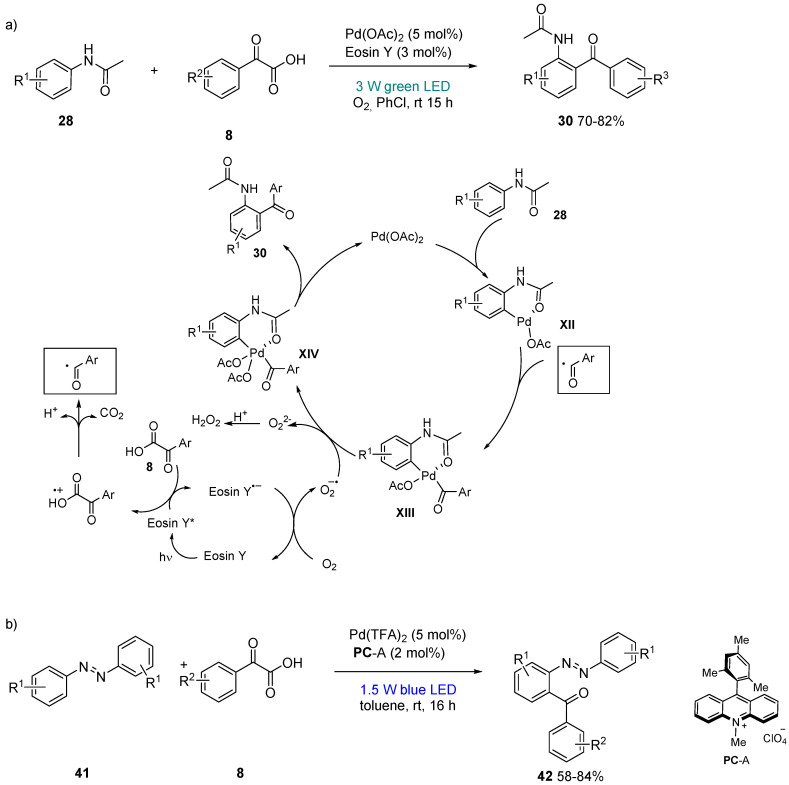
Photoredox/palladium catalyzed acylation of acetanilides and azoarenes.

To finish this section, an important contribution is depicted in Scheme 13. Recently, the combination of visible light photoredox catalysis with transition metal catalysis has attracted much attention, as it can open opportunities for new reactivity [46]. In this context, it has been shown that the acylation reaction of acetanilides **28** [47] (Scheme 13a) and azoarenes **41** [48] (Scheme 13b) can be carried out at room temperature using a combination of a photoredox catalyst with a palladium(II) catalyst. In both cases, the reaction would work on a Pd(II)/Pd(IV) catalytic cycle, analogous to the one depicted in Scheme 1. This cycle would be coupled with the corresponding photocatalytic cycle, through which the acyl radical is generated from the oxoacid **8**. The presence of the acyl radical has been supported by TEMPO trapping experiments and the presence of oxygen (air) is required. In both cases, several photoredox catalysts were screened and Eosin Y and the acridinium salt perchlorate PC-A (Fukuzumi salt) were selected as the best candidates. In both cases, yields of the ketones **30** and **42** are competitive with the ones obtained in the presence of an oxidant (Scheme 8 and Scheme 10), with the advantage of using milder conditions in the absence of an excess of oxidant. The proposed mechanism is illustrated in Scheme 13a for the reaction of acetanilides **28**. Palladium catalytic cycle would start by a C-H activation to form palladacycle **XII**, which reacts with the acyl radical to afford a Pd(III) intermediate **XIII**. A one electron oxidation via superoxide radical anion generates a Pd(IV) intermediate **XIV**. Reductive elimination leads to **30** and regenerates the Pd(II) catalyst. The photocatalytic cycle starts with the visible irradiation of Eosin Y to take it to the excited state (Eosin Y*). One electron oxidation of the ketoacid **8** generates the acyl radical, after loss of CO_2_ and Eosin Y radical anion (Eosin Y^−·^). Electron transfer by molecular oxygen regenerates Eosin Y and produces superoxide radical anion (detected by Electron Spin Resonance (ESR)), who acts as an oxidant in the palladium cycle. 

## 3. Acylation of Heteroarenes 

**Scheme 14:**
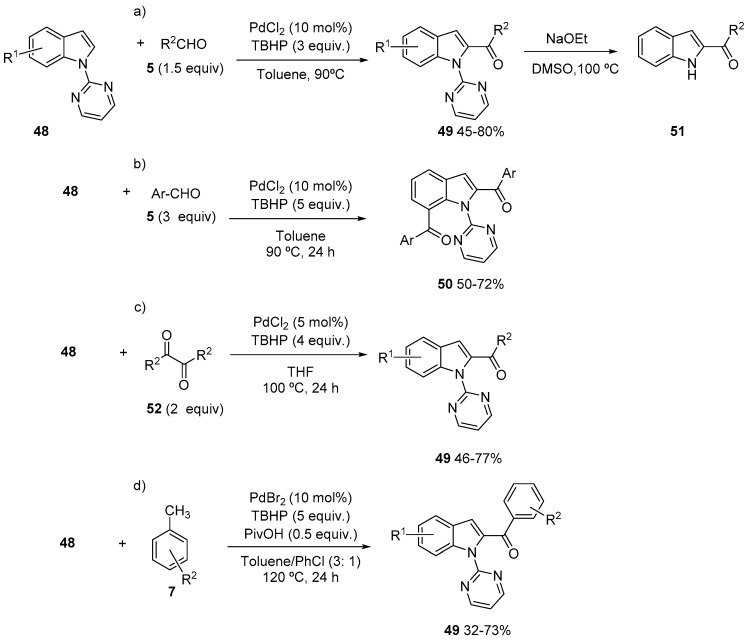
Pyrimidine-directed C-2 acylation of indoles.

The Pd(II)-catalyzed oxidative acylation of heteroarenes has been less developed than the acylation of arenes covered in the previous section. In some cases, it is possible to take advantage of the electronic properties of the heteroaromatic ring (mainly electron rich), so the C-H palladation step can be performed without the need of a directing group. However, directing groups are frequently used to overcome the electronic bias and direct the metalation to other positions in the ring. Among them, the most frequently used are nitrogen-based directing groups (2-pyridine, 2-pyrimidine). The acylation of indole derivatives has received much attention. It has been shown that the Pd(II)-catalyzed acylation of *N*-alkylindoles with aldehydes in the presence of TBHP occurs selectively at the C-3 position, through an acyl radical insertion into the initially generated 3-indoly-Pd(II) intermediate [49]. However, C-2 selective acylation of indoles has been described using 2-pyrimidine or 2-pyridine as a directing group on nitrogen and α-oxocarboxylic acids [50] and aldehydes [51]. More recently, using 2-pyrimidine as directing group, it has been shown that the acylation of indoles **48** can be achieved with both aliphatic and aromatic aldehydes **5** at the C-2 position (Scheme 14a) [52]. The directing group can be efficiently removed leading to 2-acylindoles **51**. Besides, a second metalation at C-7 can also take place, so 2,7-diacylated indoles **50** can be obtained in good yields, using a larger excess of aldehyde. If the reaction is carried out sequentially with different aldehydes **5**, non-symmetrically substituted diacylindoles **50** could also be obtained (Scheme 14 b) [52]. In a similar way, diketones **52** (Scheme 14c) [53] and toluene derivatives **7** (Scheme 14d) [54] have been used as acyl surrogates. In all cases, the oxidative formation of an acyl radical from **5**, **7** or **52** is proposed, so the mechanistic proposal would be in accordance with the general mechanism shown in Scheme 1. In all cases, high temperatures and long reaction times are required but it has been shown that the use of an acid additive (pivalic acid, Scheme 14d) increases the reaction rate, presumably by generating more electrophilic palladium species and, consequently, facilitating the C-H activation event. However, the acid may also have a detrimental effect by protonation of the substrate. Both with toluenes **7** and aromatic aldehydes **5**, the introduction of electron donating groups on the aromatic ring leads to increased yields of **49**, which would be in accordance with a more nucleophilic acyl radical. However, the opposite trend was observed with diketones **52**. Interestingly, when other directing groups, such as sulfonylpyridyl, Boc or *N,N*-dimethylcarbamoyl, were tested, no reaction took place [54], which is in contrast with related rhodium-catalyzed acylation reactions [55]. 

**Scheme 15:**
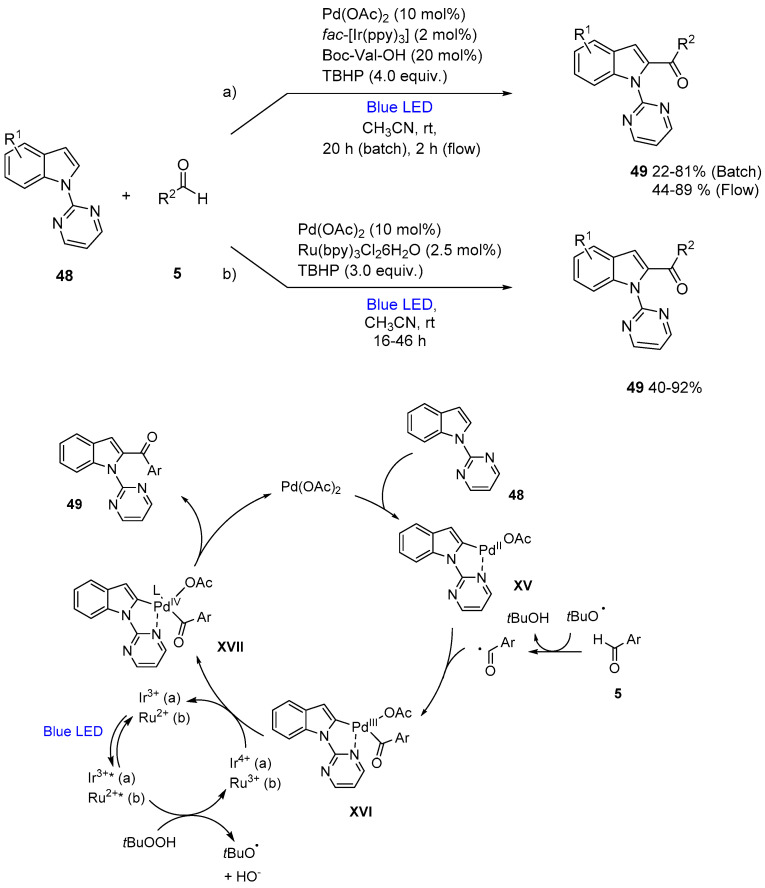
Photoredox/palladium catalyzed C-2 acylation of indoles.

Dual visible light photoredox/palladium catalysis has been also developed for the acylation of indole, using pyrimidine as directing group and aldehydes as the acyl source. Thus, indoles **48** could be efficiently acylated at room temperature in excellent yields with a wide variety of aromatic, heteroaromatic and aliphatic aldehydes **5** (Scheme 15a) [56]. The reaction was carried out both in batch and into a continuous-flow micro reactors, under blue LED light, using *fac*-[Ir(ppy)_3_] as photoredox catalyst and TBHP as the oxidant. In general, much shorter reaction times (2 h vs. 20 h), decreased catalyst loading and higher yields were obtained when the reaction was carried out in a continuous-flow reactor (Scheme 15a). Almost simultaneously, a dual catalytic system that uses a ruthenium photoredox catalyst was reported, also at room temperature with consistently good yields (Scheme 15b) [57]. As in the previously discussed examples (Scheme 13), the Pd(II)/Pd(IV) cycle for the C-H activation and acylation would be combined with the photoredox cycle but in these cases, the presence of an oxidant (TBHP) is also necessary. A mechanistic proposal is depicted in Scheme 15. In the presence of light, Ir^3+^ or Ru^2+^ are excited to Ir^3+^* or Ru^2+^*. Single electron transfer to TBHP produces the *t*-butoxy radical that, in turn, abstracts a hydrogen from the aldehyde **5** to generate the acyl radical. The addition of the acyl radical to palladium (II) intermediate **XV** generates a Pd(III) intermediate **XVI**, which is oxidized by Ru^3+^ or Ir^4+^ closing the photocatalytic cycle and generating Pd(IV) intermediate **XVII**. Reductive elimination produces **49** and regenerates de Pd(II) catalyst.

**Scheme 16:**
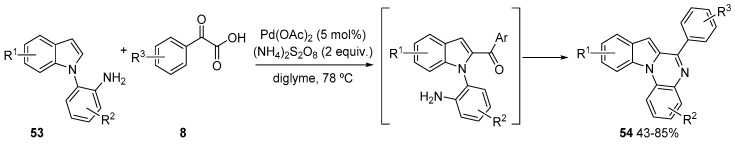
Amine directed acylation/cyclization. Access to indolo[1,2-*a*]quinazolines.

Other directing groups have also been used for C-2 acylation. As shown in Scheme 16, the amine directed palladation of indoles **53** was possible through the formation of a six-membered ring palladacycle. Kinetic isotopic effect (KIE) experiments suggested that the cleavage of the C-H would be rate determining. The acylation takes place efficiently with a variety of aromatic α-oxoacids **8**, obtaining the indolo[1,2-*a*]quinazolines **54** after condensation. The reaction takes also place in the presence of TEMPO and other radical scavengers, suggesting that an acyl radical is not involved. The authors propose an alternate Pd(II)/Pd(0) mechanism, in which the oxidant is required for the reoxidation of the palladium catalyst [58].

**Scheme 17:**
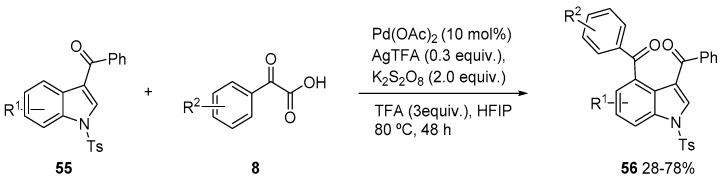
Ketone directed selective C-4 acylation of indoles.

C-4 position of indoles **55** can also be selectively acylated using a ketone carbonyl group in C-3 as directing group (Scheme 17) [59]. The metalation takes place with complete regioselectivity at the C-4 position, through the formation of a six-membered palladacycle, which would be more favorable than C-2 metalation that would imply a more strained five-membered palladacycle. The choice of the protecting group on the nitrogen atom is also critical for reactivity.

**Scheme 18:**
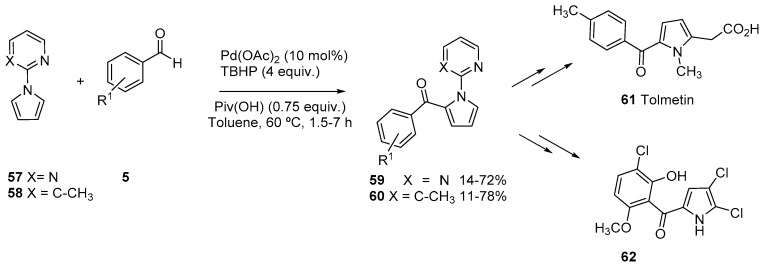
Pyrimidine and 6-methylpyridine-directed acylation of pyrroles.

In contrast with the examples discussed for indoles, only a few examples of acylation of pyrroles have been described. When 2-pyrimidine was used as directing group, mixtures of C2-acylated pyrrole and the corresponding 2,5-diacylated product were obtained in modest yields [53]. Diacylated pyrroles have also been obtained using 2-pyridine as directing group on pyrrole, under conditions optimized for indole systems [51]. However, we have recently shown that the use of 2-pyrimidine as directing group, and the use of an acidic additive (PivOH), allows the C-2 acylation of pyrrole **57** with aldehydes **5** in moderate to good yields (Scheme 18) [60]. However, in most of the cases, a minor amount of the corresponding 2,5-diacylated pyrrole was also obtained. We reasoned that a change in the directing group, introducing a substituent at C-3, could result in a steric interaction that may prevent the adoption of the required conformation to assist the second palladation. In fact, when 3-methyl-2-pyridinyl was used as directing group (**58**), monoacylated pyrroles **60** were obtained with complete selectivity. Both directing groups could be easily removed, so selected acylated pyrroles have been used as intermediates in the synthesis of Celastramycin analogues, such as **62** and in an improved synthesis of Tolmetin **61.**

**Scheme 19:**
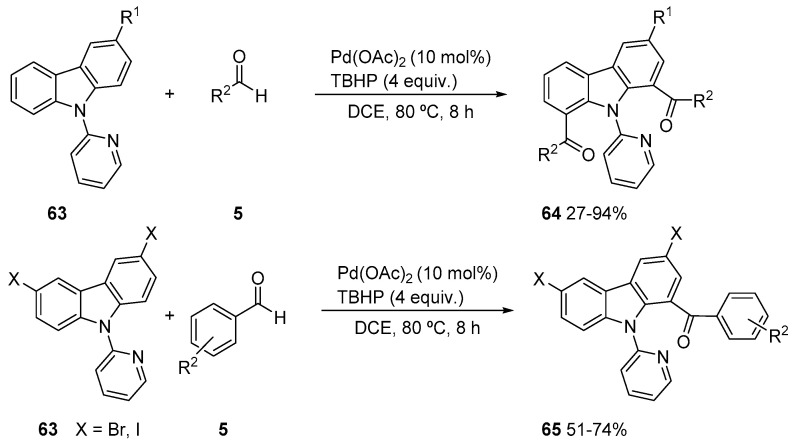
Pyridine-directed acylation of carbazoles.

2-Pyridine has been used for the acylation of carbazoles **63** (Scheme 19) [61] with various aromatic and aliphatic aldehydes **5**. When only one equivalent of the aldehyde **5** was used, a mixture of mono- and diacylated carbazoles were obtained in low yields. However, the use of 4 equivalents of **5** led to the selective formation of diacylated carbazoles **64** in generally high yields in the case of aromatic aldehydes. Lower yields were obtained with aliphatic aldehydes. Interestingly, when 3,6-dihalogenated carbazoles **63** (X = Br, I) were used, monoacylated carbazoles **65** were selectively obtained. 

**Scheme 20:**
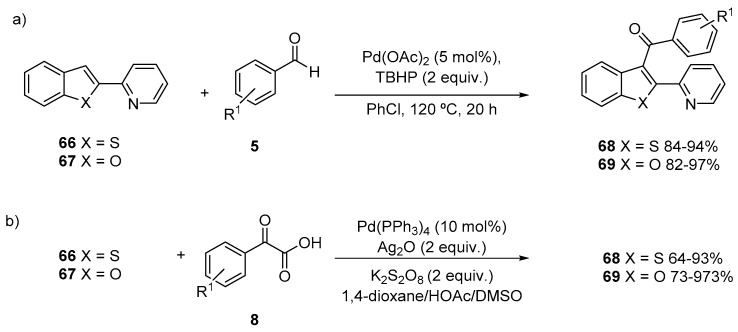
Pyridine-directed acylation of benzofurans and benzothiophenes.

2-Pyridine has also been introduced at the C-2 position of benzothiophenes **66** and benzofurans **67** to obtain the C-acylated derivatives **68** and **69** using aromatic aldehydes (Scheme 20a) [62] or α-oxoacids **8** (Scheme 20b) [63] as the acyl sources. In the latter case, a Pd(0) pre-catalyst is employed that would be oxidized *in situ*.

**Scheme 21:**
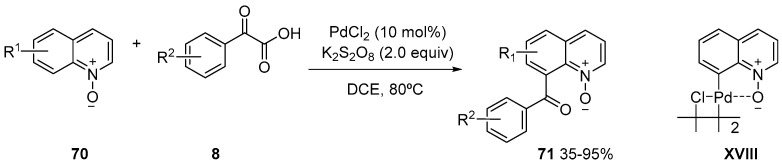
*N*-oxide directed C-8 acylation of quinolines.

The examples of heteroarene acylation shown so far involve electron rich heteroaromatic rings that, in principle, could be more easily metalated with an electrophilic Pd(II) catalyst. However, selective C-8 palladium catalyzed acylation of quinolines has also been accomplished (Scheme 21) [64] using an *N*-oxide as the directing group. The formation of an *N*-oxide chelated palladacycle would favor the regioselective acylation of quinoline *N*-oxides **70** with a variety of aromatic α-oxoacids **8**. Besides, quinolines with electron donating groups gave better yields of acylquinolines **71** than those with electron withdrawing groups. Intermolecular KIE experiments indicate that the C-H bond cleavage would be rate determining. Besides, the reaction of quinoline *N*-oxide with stoichiometric PdCl_2_ gave a chloride-bridged palladacycle dimer **XVIII**. This complex **XVIII** was reacted with an oxoacid **8** in the presence of oxidant, which suggests the formation of a five membered palladacycle in the catalytic cycle. 

## 4. Conclusions and Outlook

As has been shown through selected examples, the oxidative Pd(II)-catalyzed acylation is an effective alternative to the classical acylation methods. This methodology has been successfully applied to the acylation of arenes and heteroarenes, using different acyl sources. In most cases, a directing group is necessary to favor the initial palladation. Although highly selective reactions have been accomplished under relatively mild conditions, further development is required, also regarding the mechanistic aspects, to face selectivity problems in order to be applied in the synthesis of more complex molecules. Reactivity problems are also apparent as, in many cases, long reaction times and relatively high temperatures are required. Besides, the use aliphatic acyl equivalents, if possible, generally provides much lower yields. The use of other techniques, such as microwave (MW) irradiation or the combination of this metal catalyzed reaction with photoredox catalysis may indeed open new opportunities for development. In addition, the demand of more sustainable processes would foster the translation of homogeneous catalytic systems into heterogeneous catalysts that may be recovered and reused. Although there are interesting examples in related Pd(II)-catalyzed alkenylation reactions using metal-organic framewrks (MOFs) [65], the use of heterogeneous systems for acylation reactions is still underdeveloped. On the other hand, the use of other more abundant and less toxic 3d transition metals on [66], such as nickel [67] or cobalt [68] for these acylation reactions would be of a great importance.

## References

[1] Lei A., Shi W., Liu W., Zhang H., He C. (2017). Oxidative Cross-Coupling Reactions.

[2] Gensch T., Hopkinson M.N., Glorius F., Wencel-Delord J. (2016). Mild Metal-catalyzed C–H Activation: Examples and Concepts. Chem. Soc. Rev..

[3] Sambiagio C., Schönbauer D., Blieck R., Dao-Huy T., Pototschnig G., Schaaf P., Wiesinger T., Zia M.F., Wencel-Delord J., Besset T. (2018). Comprehensive Overview of Directing Groups Applied in Metal-Catalysed C–H Functionalization Chemistry. Chem. Soc. Rev..

[4] Jia X., Zhang S., Wang W., Luo F., Cheng J. (2009). Palladium-Catalyzed Acylation of sp^2^ C-H bond: Direct Access to Ketones from Aldehydes. Org. Lett..

[5] Banerjee A., Lei Z., Ngai M.Y. (2019). Acyl Radical Chemistry via Visible-Light Photoredox Catalysis. Synthesis.

[6] Duan P., Yang Y., Ben R., Yan Y., Dai l., Hong M., Wu Y.D., Wang D., Zhang X., Zhao J. (2014). Palladium-catalyzed Benzo[*d*]isoxazole Synthesis by C-H Activation/[4+1] Annulation. Chem. Sci..

[7] Chu J.H., Chen S.T., Chiang M.F., Wu M.J. (2015). Palladium Catalyzed Direct *Ortho* Aroylation of 2-Phenoxypyridines with Aldehydes and Catalytic Mechanistic Investigation. Organometallics.

[8] Wu X.-F. (2015). Acylation of (Hetero)Arenes through C-H Activation with Aroyl Surrogates. Chem. Eur. J..

[9] Hummel J.R., Boerth J.A., Ellman J.A. (2017). Transition-Metal-Catalyzed C−H Bond Addition to Carbonyls, Imines and Related Polarized π Bonds. Chem. Rev..

[10] Baslé O., Bidange J., Shuai Q., Li C.J. (2010). Palladium-Catalyzed Oxidative sp^2^ CH Bond Acylation with Aldehydes. Adv. Synth. Catal..

[11] Perumgani C.P., Parvathaneni S.P., Keesara S., Mandapati M.R. (2016). Recyclable Pd(II) Complex Catalyzed Oxidative sp^2^ C-H Bond Acylation of 2-Aryl Pyridines with Toluene Derivatives. J. Organomet. Chem..

[12] Li M., Ge H. (2010). Decarboxylative Acylation of Arenes with α-Oxocarboxylic Acids via Palladium-Catalyzed C-H Activation. Org. Lett..

[13] Hossian A., Manna M.K., Manna K., Jana R. (2017). Palladium-catalyzed Decarboxylative, Decarbonylative and Dehydrogenative C(sp^2^)–H Acylation at Room Temperature. Org. Biomol. Chem..

[14] Chu J.H., Chiang M.F., Li C.W., Su Z.H., Lo S.C., Wu M.J. (2019). Palladium Catalyzed Late Stage *ortho*-C-H Bond Aroylation of anilines using 4-Methoxy-2-pyridinyl as a Removable Directing Group. Organometallics.

[15] Kolle S., Batra S. (2016). β-Carboline-directed Decarboxylative Acylation of *Ortho*-C(sp^2^)–H of the Aryl Ring of Aryl(β-carbolin-1-yl)methanones with α-Ketoacids under Palladium Catalysis. RSC Adv..

[16] San Segundo M., Correa A. (2019). Pd-catalyzed Site-selective C(sp^2^)–H Radical Acylation of Phenylalanine Containing Peptides with Aldehydes. Chem. Sci..

[17] Wang Z., Tian Q., Yu X., Kuang C. (2014). Palladium-Catalyzed Acylation of 2-Aryl-1,2,3-triazoles with Aldehydes. Adv. Synth. Catal..

[18] Zhao F., Chen Z., Liu Y., Xie K., Jiang Y. (2016). Palladium-Catalyzed Acylation of Arenes by 1,2,3-Triazole-Directed C–H Activation. Eur. J. Org. Chem..

[19] Ma X., Huang H., Yang J., Feng X., Xie K. (2018). Palladium-Catalyzed Decarboxylative *N*-3-*ortho*-C–H Acylation of 1,4-Disubstituted 1,2,3-Triazoles with α-Oxocarboxylic Acids. Synthesis.

[20] Banerjee A., Bera A., Santra S.K., Guin S., Patel B.K. (2014). Palladium-catalysed Regioselective Aroylation and Acetoxylation of 3,5-Diarylisoxazole via *Ortho* C–H Functionalisations. RSC Adv..

[21] Snieckus V. (1990). Directed *Ortho* Metalation. Tertiary Amide and *O*-carbamate Directors in Synthetic Strategies for Polysubstituted Aromatics. Chem. Rev..

[22] Whisler M.C., MacNeil S., Snieckus V., Beak P. (2004). Beyond Thermodynamic Acidity: A Perspective on the Complex-Induced Proximity Effect (CIPE) in Deprotonation Reactions. Angew. Chem. Int. Ed..

[23] Park J., Park E., Kim A., Lee Y., Chi K.W., Kwak J.H., Jung Y.H., Kim I.S. (2011). Rhodium-Catalyzed Oxidative *ortho*-Acylation of Benzamides with Aldehydes: Direct Functionalization of the sp^2^ C–H Bond. Org. Lett..

[24] Meng G., Shi S., Szostak M. (2016). Cross-Coupling of Amides by N–C Bond Activation. Synlett.

[25] Laha J.K., Patel K.V., Sharma S. (2017). Palladium-Catalyzed Decarboxylative *Ortho*-Acylation of Tertiary Benzamides with Arylglyoxylic Acids. ACS Omega..

[26] Jing K., Yao J.-P., Li Z.-Y., Li Q.-L., Lin H.-S., Wang G.-W. (2017). Palladium-Catalyzed Decarboxylative *Ortho*-Acylation of Benzamides with α-Oxocarboxylic Acids. J. Org. Chem..

[27] Yu Q., Zhang N., Huang J., Lu S., Zhu Y., Yu X., Zhao K. (2013). Efficient Synthesis of Hydroxyl Isoindolones by a Pd-Mediated C-H Activation/Annulation Reaction. Chem. Eur. J..

[28] Yang L., Han L., Xu B., Zhao L., Zhou J., Zhang H. (2016). Palladium-Catalyzed C-H Bond *Ortho* Acylation/Annulation with Toluene Derivatives. Asian J. Org. Chem..

[29] Jing K., Wang X.-N., Wang G.-W. (2019). Diastereoselective Synthesis of Oxazoloisoindolinones via Cascade Pd-Catalyzed *Ortho*-Acylation of *N*-Benzoyl α-Amino Acid Derivatives and Subsequent Double Intramolecular Cyclizations. J. Org. Chem..

[30] Chan C.W., Zhou Z., Yu W.Y. (2011). Palladium(II)-Catalyzed Direct *Ortho*-C−H Acylation of Anilides by Oxidative Cross-Coupling with Aldehydes using *Tert*-Butyl Hydroperoxide as Oxidant. Adv. Synth. Catal..

[31] Wu Y., Li B., Mao F., Li X., Kwong F.Y. (2011). Palladium-Catalyzed Oxidative C−H Bond Coupling of Steered Acetanilides and Aldehydes: A Facile Access to *ortho*-Acylacetanilides. Org. Lett..

[32] Li C., Wang L., Li P., Zhou W. (2011). Palladium(II)-Catalyzed Direct *Ortho*-C−H Acylation of Anilides by Oxidative Cross-Coupling with Aldehydes using *Tert*-Butyl Hydroperoxide as Oxidant. Chem. Eur. J..

[33] Shin Y., Sharma S., Mishra N.K., Han S., Park J., Oh H., Ha J., Yoo H., Jung H.-Y., Kim I.S. (2015). Direct and Site-Selective Palladium-Catalyzed C-7 Acylation of Indolines with Aldehydes. Adv. Synth. Catal..

[34] Luo F., Yang J., Li Z., Xiang H., Zhou X. (2015). Palladium-Catalyzed C–H Bond Acylation of Acetanilides with Benzylic Alcohols under Aqueous Conditions. Eur. J. Org. Chem..

[35] Li Q.-L., Li Z.-Y., Wang G.-W. (2018). Palladium-Catalyzed Decarboxylative *Ortho*-Acylation of Anilines with Carbamate as a Removable Directing Group. ACS. Omega..

[36] Ojha S., Panda N. (2020). Palladium-Catalyzed *Ortho*-Benzoylation of Sulfonamides through C−H Activation: Expedient Synthesis of Cyclic *N*-Sulfonyl Ketimines. Adv. Synth. Catal..

[37] Das P., Biswas P., Guin J. (2020). Palladium-Catalyzed Decarboxylative *Ortho*-C(sp^2^)−H Aroylation of *N*-Sulfoximine Benzamides at Room Temperature. Chem. Asian J..

[38] Li H., Li P., Wang L. (2013). Direct Access to Acylated Azobenzenes via Pd-Catalyzed C–H Functionalization and Further Transformation into an Indazole Backbone. Org. Lett..

[39] Xiao F., Chen S., Huang H., Deng G.J. (2015). Palladium-Catalyzed Oxidative Direct *Ortho*-C–H Acylation of Arenes with Aldehydes under Aqueous Conditions. Eur. J. Org. Chem..

[40] Li Z.-Y., Li D.-D., Wang G.-W. (2013). Palladium-Catalyzed Decarboxylative *Ortho* Acylation of Azobenzenes with α-Oxocarboxylic Acids. J. Org. Chem..

[41] Li H., Li P., Tan H., Wang. L.A. (2013). Highly Efficient Palladium-Catalyzed Decarboxylative ortho-Acylation of Azobenzenes with α-Oxocarboxylic Acids: Direct Access to Acylated Azo Compounds. Chem. Eur. J..

[42] Majhi B., Ahammed S., Kundu D., Ranu B.C. (2015). Palladium-Catalyzed Oxidative C−C Bond Cleavage of α-Hydroxyketones: Application to C−H Acylation of Azoarenes and Synthesis of a Liver(X) Receptor Agonist. Asian J. Org. Chem..

[43] Wu Y., Sun L., Chen Y., Zhou Q., Huang J.W., Miao H., Luo H.B. (2016). Palladium-Catalyzed Decarboxylative Acylation of *N*-Nitrosoanilines with α-Oxocarboxylic Acids. J. Org. Chem..

[44] Zhang L., Wang Z., Guo P., Sun W., Li Y.-M., Sun M., Hua C. (2016). Palladium-catalyzed *Ortho*-acylation of *N*-Nitrosoanilines with α-Oxocarboxylic Acids: A Convenient Method to Synthesize *N*-Nitroso Ketones and Indazoles. Tetrahedron Lett..

[45] Yao J.P., Wang G.W. (2016). Palladium-catalyzed Decarboxylative *Ortho*-acylation of *N*-Nitrosoanilines with α-Oxocarboxylic Acids. Tetrahedron Lett..

[46] De Abreu M., Belmont P., Brachet E. (2020). Synergistic Photoredox/Transition-Metal Catalysis for Carbon–Carbon Bond Formation Reactions. Eur. J. Org. Chem..

[47] Zhou C., Li P., Zhu X., Wang L. (2015). Merging Photoredox with Palladium Catalysis: Decarboxylative *Ortho*-Acylation of Acetanilides with α-Oxocarboxylic Acids under Mild Reaction Conditions. Org. Lett..

[48] Xu N., Li P., Xie Z., Wang L. (2016). Merging Visible-Light Photocatalysis and Palladium Catalysis for C-H Acylation of Azo- and Azoxybenzenes with α-Keto Acids. Chem. Eur. J..

[49] Kianmehr E., Kamezi S., Foroumadi A. (2014). Palladium-catalyzed Oxidative C−H bond coupling of Indoles and Benzaldehydes: A New Approach to the Synthesis of 3-Benzoylindoles. Tetrahedron.

[50] Pan C., Jin H., Liu X., Cheng Y., Zhu C. (2013). Palladium-catalyzed Decarboxylative C2-Acylation of Indoles with α-Oxocarboxylic Acids. Chem. Commun..

[51] Yan X.-B., Shen Y.-W., Chen D.-Q., Gao P., Li Y.-X., Song X.-R., Liu X.-Y., Liang Y.-M. (2014). Palladium-catalyzed C2-Acylation of Indoles with Aryl and Alkyl Aldehydes. Tetrahedron.

[52] Kumar G., Sekar G. (2015). Pd-catalyzed Direct C2-Acylation and C2,C7-Diacylation of Indoles: Pyrimidine as an Easily Removable C–H Directing Group. RSC Adv..

[53] Li C., Shu S., Wu X., Liu H. (2015). Palladium-Catalyzed C2-Acylation of Indoles with α-Diketones Assisted by the Removable *N*-(2-Pyrimidyl) Group. Eur. J. Org. Chem..

[54] Zhao Y., Sharma U.K., Schröder F., Sharma N., Song G., Van der Eycken E.V. (2017). Direct C-2 Acylation of Indoles with Toluene Derivatives via Pd(II)-catalysed C–H Activation. RSC Adv.

[55] Zhou B., Yang Y., Li Y. (2012). Rhodium-catalyzed Oxidative C2-Acylation of Indoles with Aryl and Alkyl Aldehydes. Chem. Commun..

[56] Sharma U.K., Gemoets H.P.L., Schröder F., Nöel T., Van der Eycken E.V. (2017). Merger of Visible-Light Photoredox Catalysis and C−H Activation for the Room-Temperature C-2 Acylation of Indoles in Batch and Flow. ACS Catal..

[57] Manna K.M., Bairy G., Jana R. (2017). Dual Visible-light Photoredox and Palladium(II) Catalysis for Dehydrogenative C2-Acylation of Indoles at Room Temperature. Org. Biomol. Chem..

[58] Jiang G., Wang S., Zhang J., Yu J., Zhang Z., Ji F. (2019). Palladium-Catalyzed Primary Amine-Directed Decarboxylative Annulation of α-Oxocarboxylic Acids: Access to Indolo [1,2-*a*] Quinazolines. Adv. Synth. Catal..

[59] Zhang J., Wu M., Fan J., Xu Q., Xie M. (2019). Selective C–H Acylation of Indoles with α-Oxocarboxylic Acids at the C4 Position by Palladium Catalysis. Chem. Commun..

[60] Santiago C., Rubio I., Sotomayor N., Lete E. (2020). Selective Pd(II)-catalyzed Acylation of Pyrrole with Aldehydes. Application to the Synthesis of Celastramycin analogues and Tolmetin. Eur. J. Org. Chem..

[61] Maiti S., Burgula L., Chakraborti G., Dash J. (2017). Palladium Catalyzed Pyridine Group Directed Regioselective Oxidative C-H Acylation of Carbazoles using Aldehydes as the Acyl Source. Eur. J. Org. Chem..

[62] Zhao J., Fang H., Xie C., Han J., Li G., Pan Y. (2013). Palladium-Catalyzed C3 Acylation of Benzofurans and Benzothiophenes with Aromatic Aldehydes by Cross-Dehydrogenative Coupling Reactions. Asian J. Org. Chem..

[63] Gong W.-J., Liu D.-X., Li F.-L., Gao J., Li H.-X., Lang J.-P. (2015). Palladium-catalyzed Decarboxylative C3-Acylation of Benzofurans and Benzothiophenes with α-Oxocarboxylic Acids via Direct sp^2^ C-H Bond Activation. Tetrahedron.

[64] Chen X., Cui X., Wu Y. (2016). C8-Selective Acylation of Quinoline *N*-Oxides with α-Oxocarboxylic Acids via Palladium-Catalyzed Regioselective C−H Bond Activation. Org. Lett..

[65] Cirujano F.G., Leo P., Vercammen J., Smolders S., Orcajo G., De Vos D.E. (2018). MOFs Extend the Lifetime of Pd(II) Catalyst for Room Temperature Alkenylation of Enamine-Like Arenes. Adv. Synth. Catal..

[66] Gandeepan P., Muüller T., Zell D., Cera G., Warratz S., Ackermann L. (2019). 3d Transition Metals for C−H Activation. Chem. Rev..

[67] Yang K., Zhang C., Wang P., Zhang Y., Ge H. (2014). Nickel-Catalyzed Decarboxylative Acylation of Heteroarenes by sp^2^ C−H Functionalization. Chem. Eur. J..

[68] Yang K., Chen X., Wang Y., Li W., Kadi A.A., Fun H.-K., Sun H., Zang Y., Li G., Lu H. (2015). Cobalt-Catalyzed Decarboxylative 2-Benzoylation of Oxazoles and Thiazoles with α-Oxocarboxylic Acids. J. Org. Chem..

